# Prevalence and clinical presentation of long COVID in children: a systematic review

**DOI:** 10.1007/s00431-022-04600-x

**Published:** 2022-09-15

**Authors:** Roberta Pellegrino, Elena Chiappini, Amelia Licari, Luisa Galli, Gian Luigi Marseglia

**Affiliations:** 1 Department of Health Sciences, Section of Pediatrics, University of Florence, Florence, Italy; 2 Pediatric Infectious Disease Unit, Anna Meyer Children’s University Hospital, Florence, Italy; 3 Department of Clinical, Surgical, Diagnostic, and Pediatric Sciences, University of Pavia, Pavia, Italy; 4 Pediatric Clinic, Fondazione IRCCS Policlinico San Matteo, Pavia, Italy

**Keywords:** COVID-19, SARS-CoV-2, Post COVID, Sequelae, Children

## Abstract

A systematic literature review was conducted up to 15th February 2022 to summarize long COVID evidence and to assess prevalence and clinical presentation in children and adolescents. Articles reporting long COVID prevalence and symptoms based on original data in the paediatric population were included. Case series quality was assessed through the JBI Critical Appraisal Checklist. For observational studies, adherence to STROBE checklist was evaluated. Twenty-two articles were included: 19 observational studies (12 cohort/7 cross-sectional) and 3 case series. Nine studies provided a control group. We found a high variability in terms of prevalence (1.6–70%). The most frequently reported symptoms were fatigue (2–87%), headache (3.5–80%), arthro-myalgias (5.4–66%), chest tightness or pain (1.4–51%), and dyspnoea (2–57.1%). Five studies reported limitations in daily function due to long COVID. Alterations at brain imaging were described in one study, transient electrocardiographic abnormalities were described in a minority of children, while most authors did not evidence long-term pulmonary sequelae. Older age, female sex, and previous long-term pathological conditions were more frequently associated with persistent symptoms.

*Conclusion*: Long COVID evidence in children is limited, heterogeneous, and based on low-quality studies. The lockdown consequences are difficult to distinguish from long COVID symptoms. High-quality studies are required: WHO definition of long COVID should be used, controlled clinical studies should be encouraged, and the impact of new variants on long COVID prevalence should be investigated to ensure an objective analysis of long COVID characteristics in children and a proper allocation of healthcare system resources.

**Table:** 

**What is Known:** *• Children rarely develop a severe respiratory disease in the acute phase of COVID-19.* *• A limited number of patients develop a multisystem inflammatory condition that can lead to multiorgan failure and shock.*	
**What is New:** *• Persistent symptoms after SARS-CoV-2 infection are reported in children and limitations in daily function due to long COVID symptoms affect school attendance.* *• Functional complaints of post-acute COVID are difficult to be distinguished from those due to social restrictions.*

**Supplementary Information:**

The online version contains supplementary material available at 10.1007/s00431-022-04600-x.

## Introduction

The challenges of coronavirus-associated acute respiratory disease called coronavirus disease 19 (COVID-19) are now extending to its long-term sequelae. Since the beginning of the severe acute respiratory syndrome coronavirus type 2 (SARS-CoV-2) [1] pandemic outbreak, evidence of persisting symptoms has emerged in adults with a prevalence of long COVID up to 80% [2]. The range of symptoms is extensive and the most common reported are fatigue, headache, attention disturbance, hair loss, and dyspnoea [2]. Several health organizations have issued different definitions of this new syndrome in adults, as reported in Table 1 [3–5]. Recently, a research definition of long COVID in children has been derived from a Delphi process and it is reported in Table 2 [6].

**Table 1:** Long COVID definitions in adults

		**Duration of symptoms**	**Previous history**	**Other criteria**
**National Institute of Health and Care Excellence (NICE)** [3]	Ongoing symptomatic COVID-19	4 to 12 weeks	Acute COVID-19	Persistence of symptoms
	Post COVID-19 syndrome	Over 12 weeks	Acute COVID-19	Persistence of symptoms
**Center for Disease Control and Prevention (CDC)** [4]		4 weeks or more after the infection	SARS-CoV-2 infection	
**World Health Organization (WHO)** [5]		At least 2 months	Probable or confirmed SARS-CoV-2 infection	- Symptoms presenting 3 months after the onset of COVID - Cannot be explained by an alternative diagnosis - Impact on everyday functioning - Symptoms may continue or develop after the infection - May fluctuate or relapse over time

**Table 2:** Long COVID definition in children

	**Duration of symptoms**	**Previous history**	**Other criteria**
**Research definition of long COVID in children and youths aligned to WHO definition** [6]	At least 12 weeks after initial testing	Confirmed SARS-CoV-2 infection (PCR, lateral flow antigen test, or antibody test)	- One or more persisting physical symptom - Cannot be explained by an alternative diagnosis - Impact on everyday functioning - Symptoms may continue or develop after the infection - May fluctuate or relapse over time

Children rarely develop a severe respiratory disease in the acute phase of COVID-19, though a limited number of patients exhibits a well-defined multisystem inflammatory condition, that can lead to multiorgan failure and shock, known as paediatric inflammatory multisystem syndrome temporally associated with SARS-CoV-2 (PIMS-TS) [7] or multisystem inflammatory syndrome in children (MIS-C) [8].

Since October 2020, parents’ concerns about persistent symptoms in children months after the acute SARS-CoV-2 infection have been emerging [9]. In November 2020, a case series from Sweden described a group of five girls with long COVID [10]. Since then, studies regarding long COVID in the paediatric population are accumulating although high variability in terms of definition, prevalence, and symptoms has been reported [11]. Therefore, we performed a systematic review of the literature to summarize the current evidence regarding this emerging condition in children, with a focus on prevalence and clinical presentation.

## Methods

### Design

A systematic review of the literature was performed according to the Preferred Reporting Items for Systematic Reviews and Meta-analyses (PRISMA) guideline recommendations [12]. The research was conducted through MEDLINE by PubMed and MedRxiV, for articles available up to 15 February 2022. References of all relevant articles were also evaluated, and pertinent articles were included. Search terms, limited to Title or Abstract, were as follows: “post-acute COVID-19,” “long COVID-19,” “SARS-Cov2,” “sequelae,” “COVID-19,” “children,” “child,” “paediatrics.”

### Inclusion and exclusion criteria

The research was restricted to English language. Articles reporting long COVID prevalence and symptoms based on original data in paediatric population were included independently from the study design. Review articles, commentaries, editorials, and letters to the author with no original data were excluded. Sample dimension was not an exclusion criterion. Studies concerning PIMS-TS were excluded, except where the number of patients with PIMS-TS was minimal [13, 14].

### Data extraction

Duplicate publications were removed, then two authors separately (RP and EC) checked the titles and abstracts and removed irrelevant studies according to the inclusion and exclusion criteria. Articles were categorized as cohort studies or case series and, according to the source of information, as based on surveys or questionnaires or on clinician-assessed data. From each study information about children population included, test used for SARS-CoV-2 infection diagnosis, follow-up time, long COVID definition, and clinical presentation were extracted. Studies including a minimal number of patients with PIMS-TS were included, and the prevalence of persistent symptoms was recalculated after excluding PIMS-TS cases for the sake of comparability.

### Quality assessment

For observational studies, adherence to Strengthening the Reporting of Observational Studies in Epidemiology (STROBE) recommendations [15] was assessed. Case series quality was evaluated using the Joanna Briggs Institute (JBI) Critical Appraisal Checklist for Case Series [16].

### Ethics

Ethics approval was not required for the systematic review component of this study.

## Results

### Study characteristics and quality

**Fig. 1:**
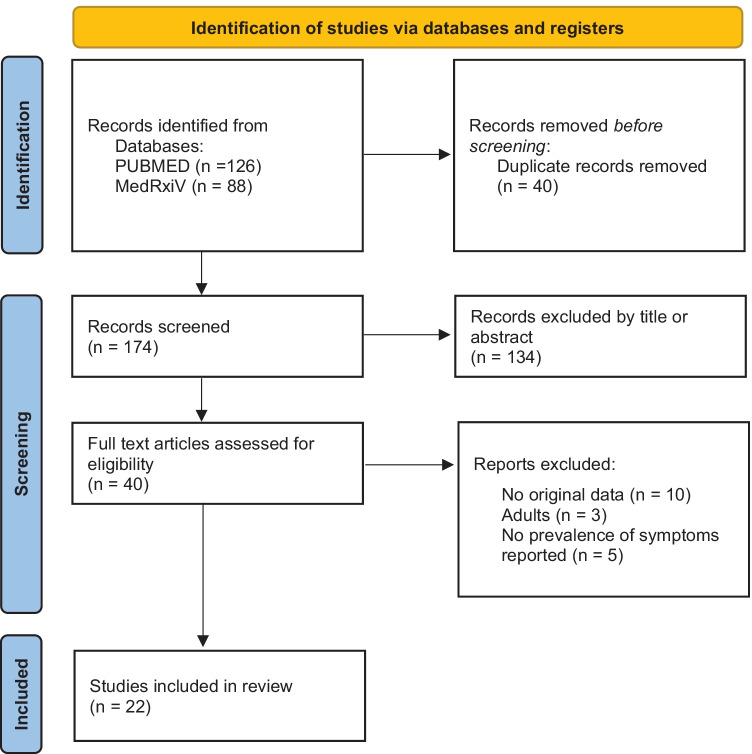
Flow diagram of literature search and data extraction

**Fig. 2:**
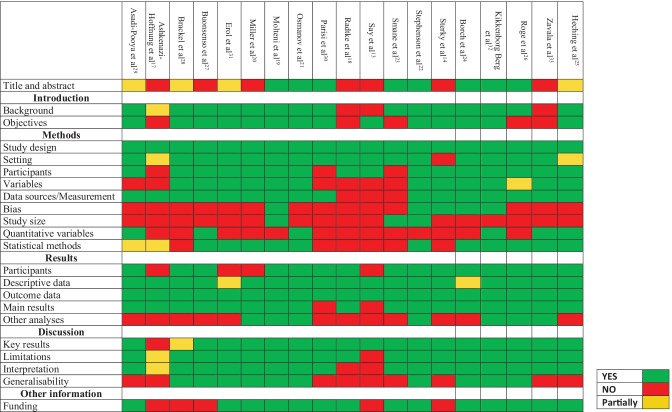
Adherence to STROBE recommendations

**Fig. 3:**
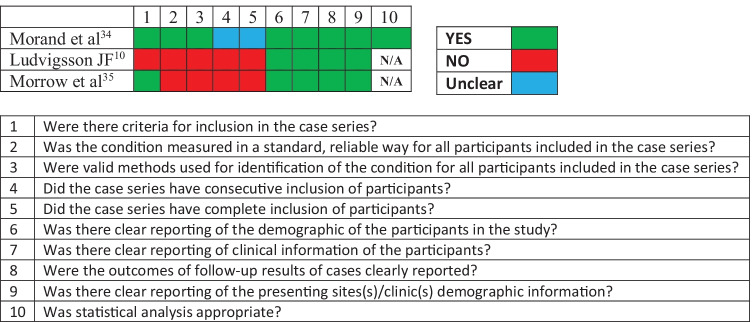
Case series quality assessment

Overall, 214 articles have been initially retrieved, and after screening and selection, 22 have been included in the review (Fig. 1). The types of studies were as follows: 12 cohort studies (8 prospective [13, 14, 17–22], 3 retrospective [23–25], and 1 ambidirectional [26]), 7 cross-sectional studies [27–33], and 3 case series [10, 34, 35]. Seven studies relied on direct assessed data [13, 17, 23, 25, 31, 34, 35], including one study with a control group [31]. Fifteen studies were based on interviews or questionnaires, of these 2 were directed to paediatricians [28, 30] and 13 to caregivers or patients [10, 14, 18–22, 24, 26, 27, 29, 32, 33] among these 8 provided a control group [18–20, 22, 24, 26, 32, 33]. The median age of children ranged from 9.16 [31] to 17.6 years [32]. As described in Tables 3 and 4, terms and definitions were quite variable. The most frequently used definition relied on symptoms persisting more than 4 weeks from acute infection or hospital admission [13, 19, 20, 24, 26, 27, 31, 34]. However, other definitions used varied from symptoms persisting over 2 months [10, 19, 32] to 5 months [21]. Follow-up time ranged from 4 weeks [34] to 13 months [24]. Adherence to STROBE recommendations for observational studies and quality assessment of case series is reported in Figs. 2 and 3, respectively. The excluded studies and the PRISMA checklist are provided in the Appendix.

**Table 3:** Studies based on clinician-assessed data

Author	Age years	Type of study	Setting	Country	Children	Diagnostic test	Long COVID (*N*)	Long COVID (%)	Follow-up	Definition
Erol et al. [31]	Median age 9.16 IQR 10.88–17.92	Cross-sectional study Control group	Inpatients and outpatients SC HC	Turkey	121 COVID-19 95 controls** to compare instrumental cardiac findings	Not known	45	37.1%	Mean 5.6 m	Symptoms persisting at least 4 w after infection
Ashkenazi-Hoffnung et al. [17]	Mean age 12 SD 5 y	Prospective cohort study	Inpatients and outpatients SC HC	Israel	90	PCR Serological test	N/A	N/A	At least 4 m	Not expressed
Say et al. [13]	Median age 3 years (IQR 1–8)	Prospective cohort study	Inpatients and outpatients SC HC	Australia	151 149 After excluding PIMS-TS	Not known	12 10 After excluding PIMS-TS	8% 6.7% After excluding PIMS-TS	6 m	Symptoms lasting over 4 w
Smane et al. [23]	Median age 12 y (IQR 8–15)	Retrospective cohort study	Outpatients SC HC	Latvia	92	PCR	47	51%	3 m	Persistence of symptoms at least 1 m after infection
Heching et al. [25]	Median age 14.4 y (range 1–18 y)	Retrospective cohort study	Outpatients SC HC	US	82	PCR or antigen test	53	65%	44.5 ± 36.2 d	Prolonged symptoms following acute infection
Morrow et al. [35]	4–18 y	Case series	Outpatients SC HC	US	8	Clinical diagnosis 4 Serological test 1 PCR 4	8	N/A	Mean 7.2 m Range 2–11 m	Persistence of symptoms after acute infection
Morand et al. [34]	Mean age 12 y [range 10–13 y]	Case series	N/A SC HC	France	661 with SARS-CoV-2 infection	Clinical 4 Serological test 2 PCR 1	7	1.6%	4 w	Persisting symptoms more than 4 w from the acute infection without symptom-free interval

*IQR* interquartile range, *N* number, *m* months, *SD* standard deviations, *y* years, *SC* single centre, *MC* multi-centre, *CW* community-wide, *HC* health/hospital-centre, *PCR* polymerase chain reaction, *N/A* not applicable, *PIMS-TS* paediatric inflammatory multisystem syndrome temporally associated with SARS-CoV-2, *US* United States of America, *d* days, *w* weeks

### Reported prevalence of long COVID in paediatric studies

**Fig. 4:**
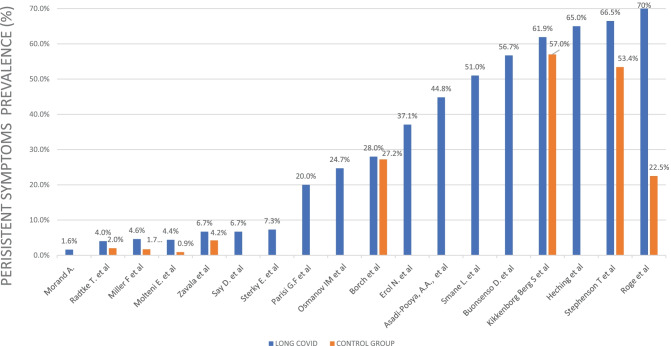
Prevalence of long COVID children reported. Studies with a sample restricted only to long COVID patients are not represented for the sake of comparability

The prevalence of long COVID varies notably from 1.6 [34] to 70% [26] (Fig. 4). The lowest was reported in a French case series describing 7 cases of long COVID out of 661 children with a positive diagnosis of COVID-19 [34]. The highest prevalence was found in a Latvian study reporting ongoing symptoms after 4 weeks in 70% of the positive cohort [26]. A cross-sectional Italian study, based on the ISARIC questionnaire [36] to caregivers, showed a similar prevalence of 58.1% of children with persisting symptoms after 4 weeks from the acute infection. By excluding 3 patients diagnosed with PIMS-TS, the long COVID prevalence dropped to 56.7% [27]. The latter prevalence was consistent with data from a study based on clinical standardized examination in 92 outpatients at a median follow-up time of 55 days after acute COVID-19 [23]. Conversely, in a subsequent study, according to most of the interviewed Italian paediatricians, the persistence of symptoms after COVID-19 was less than 20% [30].

### Clinical picture in children and adolescents

**Fig. 5:**
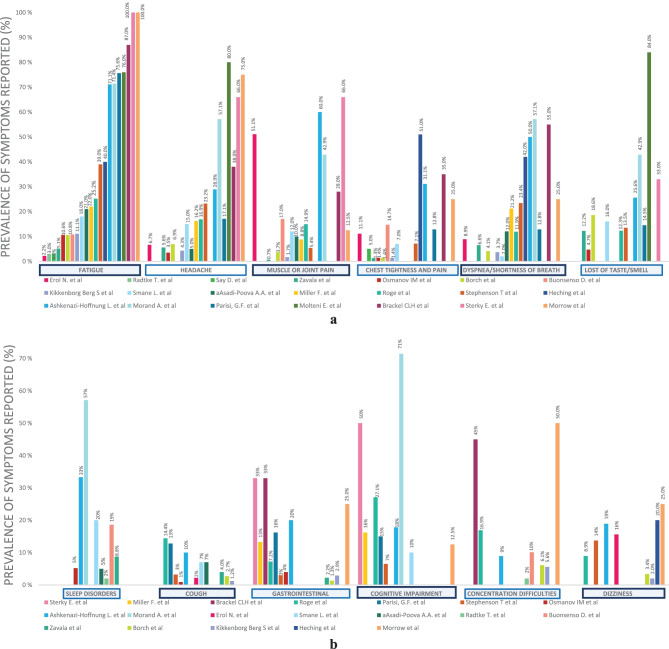
**a** Most frequently reported symptoms. **b** Other symptoms reported

The clinical spectrum assessed across studies varied notably. The most frequently reported symptoms were the following: fatigue (2 [31] to 87% [28]), headache (3.5 [21] to 80% [19]), muscle or joint pain (0.7 [33] to 66% [14]), chest tightness or pain (1.3 [33] to 51% [25]), dyspnoea (2 [23] to 57.1% [34]), and taste or smell impairment (4.7 [21] to 84% [19]) (Fig. 5). Limitation in daily function affecting school attendance was reported in 5 studies [14, 17, 28, 29, 32] ranging from 10.5 [32] to 58.9% [17]. The median symptom burden was 8 symptoms over the entire illness [19] with a tendency to decrease over the time [19, 32]. According to Osmanov et al., headache and sleep disorders tend to decline slower than the others [21].

### Results from controlled studies

Eight of the studies included in our review provided a control group [18–20, 22, 24, 26, 32, 33]. One of the first matched cohort studies in the paediatric population was the CLoCK study [37]. Preliminary results of the latter study showed that 3 months after acute infection, 66.5% of positive children had at least one symptom, in line with the negative control group where 53.4% of children had symptoms at the same timing [22]. The difference increased by comparing children with 3 or more symptoms: 30.3% among test-positive and 16.2% among test-negative [22], suggesting a higher burden of symptoms in the case group, as seen also in two Danish studies [24, 32].

Molteni et al. identified two classes of children based on illness duration, termed LC28 for duration over 28 days and LC56 over 56 days [19]. The observed prevalence was respectively 4.4% and 1.8% among children with a history of SARS-CoV-2 infection, whereas only 0.9% of the children in the control group complained of symptoms lasting over 28 days [19]. These results are consistent with the ones derived from the largest cohort to date, in which children with a history of SARS-CoV-2 infection reported persistent symptoms more frequently than the control group with a percentage difference of 0.8% [24].

A Latvian study compared children with previous SARS-CoV-2 infection to children with other non-SARS-CoV-2 infections stating that symptoms persistence is more evident with COVID-19 than any different infection [26]. On the other hand, no significant difference has been found in a Swiss cohort that described symptoms lasting over 4 weeks in 4% of seropositive and over 12 weeks in 9%, comparable to the prevalence in the seronegative group (respectively 2% and 10%) [18].

Among controlled studies, the long COVID clinical spectrum is undefined. Stephenson et al. described tiredness (23% vs 14.2%) and headache (39% vs 24.2%) as more frequently reported within the case group, and no difference in the distribution of mental health and well-being scores was found between the two groups [22]. Similarly, headache and concentration difficulties, along with fatigue, were the most frequent symptoms in the case group of the LongCOVIDKidsDK study [32]. Besides, in a nationwide matched cohort study, fatigue, anosmia, and ageusia were significantly associated with previous SARS-CoV-2 infection, whereas concentration difficulties, headache, arthro-myalgias, and gastrointestinal symptoms were more frequent in the control group [24]. Both the latter studies reported a better quality of life in children with a previous history of SARS-CoV-2 infection. The authors speculate that the lower sense of well-being in uninfected children could reflect the effects of social restrictions [24, 32].

**Table 4:** Questionnaire and survey-based studies

Author	Age years	Type of study	Setting	Country	Children	Diagnostic test	Long COVID (*N*)	Long COVID (%)	Follow-up	Definition
Stephenson et al. [22]	11–17	Prospective cohort study Control group	Inpatients and outpatients CW	England	6804 tested 3065 positives 3739 negatives	PCR	N/A	66.5% PCR positive 53.4% PCR negative	3 m	Presence of symptoms at 3 months post-testing
Molteni et al. [19]	5–17	Prospective cohort study Control group	Inpatients and outpatients SC CW	UK	1734 positives 1734 negatives	PCR Serological test	Positive: LC28 77 LC56 25 Negative with symptoms > 28 d: 15	Positive: LC28 4.4% LC56 1.8% Negative with symptoms > 28 d: 0.9%	N/A	Symptoms lasting > 28 days LC28 > 56 days LC56
Radtke et al. [18]	Median age 11 IQR 9–13	Prospective cohort study Control group	Inpatients and outpatients CW	Switzerland	109 positives 1246 negatives	Serological test	Symptoms over 4 w: 10 positive 121 negative Over 12 w: 4 positive 28 negative	Symptoms over 4 w: 9% positive 10% negative Over 12 w: 4% positive 2% negative	6 m	Symptoms lasting over 12 w
Miller et al. [20]	0–17	Prospective cohort study Control group	Outpatients MC CW	UK	175 positives 4503 negatives	PCR (62.9%) Serological test (26.9%) Both (10.3%)	80	1.7% overall 4.6% of positive	3 m	Symptoms lasting over 4 w not explained by alternative diagnosis
Osmanov et al. [21]	Median age 10.4 y (IQR 3–15) Range 2 d–18 y	Prospective cohort study	Inpatients SC HC	Russia	518	PCR	128	24.7%	Median 268 d (IQR 233–284)	Symptoms present at the time of follow-up interview and lasting over 5 months
Sterky et al. [14]	0–18	Prospective cohort study	Inpatients MC HC	Sweden	55 53 After excluding PIMS-TS	PCR	6 4 After excluding PIMS-TS	10% 7.5% After excluding PIMS-TS	At least 4 m after admission (median 219 days, range 123–324 days)	Symptoms lasting at least 4 months after admission
Buonsenso et al. [27]	11.4 SD 4.4	Cross-sectional study	Outpatients and inpatients SC HC	Italy	129	PCR	75	58.1%	Mean 162.5 d	Symptoms persisting over 30 d
Parisi et al. [30]	N/A	Cross-sectional study Online survey to paediatricians	Outpatients and inpatients HC	Italy	267 paediatricians	N/A	N/A	< 20% according to 97.3% of paediatricians	N/A	Persistence of symptoms after recovery (no timing expressed)
Brackel et al. [28]	Median age 13 (IQR 9–15) range 2–18	Cross-sectional study Survey to paediatricians	Inpatients HC	Netherlands	78% of Dutch paediatric department	PCR 47 (52.8%) Serology test 31 (34.8%) Clinical 34 (38.2%)	89	N/A	N/A	Symptoms persisting over 12 w and not explained by alternative diagnosis
Asadi-Pooya et al. [29]	6–17 (mean 12.3 SD 3.31)	Cross-sectional study	Inpatients MC HC	Iran	58	PCR	26	44.8%	3 m	Symptoms persisting at least 3 months not present before acute COVID-19
Ludvigsson [10]	9–15 mean age 12	Case series	Inpatients and outpatients SC HC	Sweden	5	Clinically diagnosed	5	N/A	N/A	Symptoms lasting over 2 months
Borch et al. [24]	Mean age 12 y (range 6–17)	Retrospective cohort study Control group	CW	Denmark	Case group 15041 Control group 15080	PCR	Case group (6–17 y) 3374 out of 12065 Control group (6–17 y) 2245 out of 8248	Case group (6–17 y) 28% Control group (6–17 y) 27.2% True long COVID prevalence estimated 0.8%	4 w–13 m	Symptoms lasting at least 4 weeks after SARS-CoV-2 infection
Kikkenborg Berg et al. [32]	Median age 17.6 y Range 16.5–18.6 y	Cross-sectional study Control group	CW	Denmark	Case group 6630 Control group 21640	PCR	Case group 3159 Control group 12340	Case group 61.9% Control group 57%	12 m	At least one symptom lasting more than 2 m
Roge et al. [26]	10 y; IQR 5–14 y; range 1 m–18 y	Ambidirectional cohort study Control group	Inpatients and outpatients SC HC	Latvia	Case group 236 Control group 142	PCR or serological test	Case group 152 Control group 32	Case group 70% Control group 22.5 Estimated long COVID prevalence in Latvian children 1.09%	73.5 d IQR 43–110 d	Symptoms experienced at least one m after acute illness
Zavala et al. [33]	Median age 10 (range 0–16)	Cross-sectional study Control group Random selection of cases and controls	Inpatients and outpatients SC CW	England	Case group 472 Control group 387	PCR	Case group 21/320 Control group 6/154	Case group 6.7% Control group 4.2%	3 m	Symptoms experienced at least 5 times at 1 month after SARS-CoV-2 infection

*N* number, *SC* single centre, *MC* multi-centre, *CW* community-wide, *HC* health/hospital-centre, *PCR* polymerase chain reaction, *N/A* not applicable, *m* months, *UK* United Kingdom, *d* days, *LC28* long COVID with symptoms persisting over 28 days, *LC56* long COVID with symptoms persisting over 56 days, *w* weeks, *IQR* interquartile range, *PIMS-TS* paediatric inflammatory multisystem syndrome temporally associated with SARS-CoV-2, *SD* standard deviations, *y* years, *ISARIC* International Severe Acute Respiratory and Emerging Infection Consortium

### Alterations in imaging and function tests in long COVID children

The persistence of long COVID symptoms has been associated with a hypometabolic pattern at positron emission tomography (PET) with 2-[18F]-fluorodeoxyglucose (FDG) of the brain, involving bilateral medial temporal lobes, brain-stream, cerebellum, and the right olfactory gyrus in 7 French children with long COVID [34].

Data regarding possible cardiac involvement are contrasting. Erol and colleagues described a statistically significant difference in systolic blood pressure, left ventricular posterior wall diameter, relative wall thickness, and tricuspid annular plane systolic excursion values between children with a history of SARS-CoV-2 infection and controls [31]. In an Israelian prospective cohort study, no echocardiographic alterations were documented in long COVID children, though lower performance at an exercise stress test was noted suggesting some degree of chronotropic incompetence [17]. Electrocardiographic (ECG) abnormalities were described in a minority of COVID-19 outpatients, and none of the subjects affected had echocardiographic alterations. The ECG abnormalities resolved over time and were not associated with severity of acute disease [25].

A mild obstructive reversible pattern at lung function test was evidenced in nearly half the children in the Israelian cohort [17], whereas no long-term pulmonary sequelae were evidenced using lung ultrasound [38, 39] and pulmonary function tests [39, 40] in 3 studies [38–40].

### Risk factors for long COVID in children

In the CLoCK study cohort, in both positive and negative groups, those with multiple symptoms were more likely to be female, adolescent, and to have poorer baseline physical and mental health status [22]. The same group of children was more likely to report problems with mobility, self-care, usual activities, and pain/discomfort after acute COVID-19 [22].

Older age as a risk factor for persistent symptoms after SARS-CoV-2 infection has been reported in 9 studies [17, 19–21, 23, 24, 26, 29, 32]. As concerns sex, in a Danish matched cross-sectional study, female subjects were more prone to show symptoms lasting more than 2 months than males, both in the case and control groups [32], whereas according to Roge et al., long COVID symptoms were more frequent among female patients, with the most significant difference in cognitive and neurological sequelae [26]. Furthermore, allergic disease [21] and previous long-term conditions [20] have been identified as possible risk factors for long COVID [20, 21].

Overweight has been described as a long COVID risk factor in adults [17]. Among studies included in our review, no statistical significant difference in terms of body mass index (BMI) was found between children reporting persistent symptoms and controls [17, 31]. Recently, Bloise et al. described obesity as a potential risk factor for long COVID syndrome also in the paediatric age [41].

No correlation between acute illness severity and duration of symptoms was noticed [27, 31], except in one study comprising only inpatients in which intensive care unit (ICU) admission was associated with long COVID [29].

### Management and follow-up of children with long COVID

The need of rehabilitation plans for long COVID patients in adults has been claimed [42], whereas the effects of this syndrome in children are unclear and data on follow-up and management are scarce. However, according to Dutch paediatricians, 29% of children with suspected long COVID required a multidisciplinary approach comprising physiotherapy and psychologist support [28]. In Italy, 86% of paediatricians stated that in their area, no reference centre dedicated to the assistance of the child recovering from COVID was available [30].

## Discussion

In the present systematic review, 7 studies [13, 17, 23, 25, 31, 34, 35] with clinical data (including 549 children with history of SARS-CoV-2 infection) and 15 studies [10, 14, 18–22, 24, 26–30, 32, 33] based on interviews or questionnaires (including 28227 children with history of SARS-CoV-2 infection) were retrieved and analysed. Data are difficult to compare due to the large inter-study variability in terms of study design, follow-up timing, and definitions of long COVID which results in different inclusion criteria. The final picture is a broad discrepancy in prevalence both for symptoms and long COVID overall. The considerable variability of prevalence and symptoms burden could indicate that studies are assessing different diseases, suggesting the urge for a harmonized case definition. Fatigue, headache, arthro-myalgia, shortness of breath, and alteration of smell or taste appear to be the most common symptoms. According to the WHO definition, the impact on everyday functioning is crucial to define long COVID. Interestingly, most of the studies relied solely on the persistence of symptoms and only five studies reported a limitation in daily function imputable to long COVID [14, 17, 28, 29, 32]. It is important to underline that most of the studies were based on proxy-reported information while clinician-assessed data were scant. Adolescent age, pre-existing long-term pathological conditions, and allergic disease have been identified as potential risk factors for persistent symptoms after acute illness [17, 19–24, 26, 29, 32]. However, a critical appraisal is necessary to understand these findings, as an example, younger children are less likely to be able to consistently report symptoms of relevance and these could lead to an underestimation of symptom prevalence in this age class. Since most of the data are derived from online surveys, a recall bias and selection bias must be considered, as symptomatic people could be more prone to participate and the answers might not be accurate.

Interestingly, persisting symptoms were described also in children with previous mild or asymptomatic COVID-19 and no correlation between the severity of acute illness and long COVID has been noted [27, 31].

Furthermore, it is unclear whether persisting symptoms are related to viral infection itself or they express the effects of pandemic, lockdown, and school suspension on children. Lockdown and social limitation negatively impacted on children and adolescent mental health [43]. This fact may explain why no statistical difference between seropositive and seronegative populations has been found in neurocognitive, pain, and mood symptoms [44]. Two studies reported better quality of life in SARS-CoV-2 infected children than controls, and the lower sense of well-being in uninfected children could reflect the psychological implications of the pandemic [24, 32]. Given that a control group is mandatory to understand the results to the fullest.

When a control group was provided, patients with a history of SARS-CoV-2 infection were more prone to show higher prevalence of persistence of symptoms [19, 20, 24, 26, 32, 33, 37], except in one study based on a small sample [18] (Fig. 4). Notably, the prevalence of symptoms declined over time, with headache and sleep disorders declining slower, which could be driven by a psychological mechanism [21]. Since the outbreak of the SARS-CoV-2 pandemic, several variants of concern have been identified. It seems that omicron cases are less likely to experience long COVID compared with delta cases in adults [45]. Currently, data on children and youths are lacking.

The symptoms observed affect cardio-respiratory, gastrointestinal, and neurological systems, and rehabilitation and psychologist support are needed [28]. Therefore, a multidisciplinary approach appears necessary to sustain children and adolescents. NICE guidelines recommend investigation in people presenting with new or ongoing symptoms 4 weeks or later after acute COVID-19, and these include a full blood count, kidney and liver function tests, a C-reactive protein test, and an exercise tolerance test [3]. Currently, no structured follow-up has been set and reference centres for paediatric population are lacking [30].

The mechanisms underlying post COVID condition are not clearly defined; however, several pathogenesis models have been put forward. One of the most supported hypotheses is based on the persistence of the virus or a virus component [46]. Several studies have demonstrated a prolonged SARS-CoV-2 shedding in the respiratory tract, faeces, and intestinal biopsies, even in asymptomatic patients [47, 48]. This could lead to an exacerbated immune response resulting in increased levels of proinflammatory cytokines, including interleukin (IL)-6, IL-1β, and TNF [49, 50]. A persistent proinflammatory state could explain organ damage and prolonged symptoms, such as fatigue, headache, and smell impairment [46, 48]. Moreover, several types of autoantibodies are produced during SARS-CoV-2 infection due to a molecular mimicry mechanism between self-antigens and spike epitopes [51]. Autoantibodies against G-protein coupled receptors (GPCRs) have been associated with post COVID-19 condition. Since GPCRs can alter the neuronal and vascular process, the autoantibody production could explain some of the neurological and cardiovascular symptoms in patients with long COVID [48].

### Limitations

Our review may have limitations, including that some articles might have been missed. Considering that the literature regarding long COVID is rapidly increasing, a continuous updating of evidence is mandatory. Methodological issues were frequent among the included studies: matched cohort studies were limited, rarely a comparison with other viral illness was provided, and most of the data were based on questionnaire-based studies. Symptoms prevalence mainly relies on self-reporting and online surveys; hence, recall and selection biases must be considered. Furthermore, most of the studies included in our review were published prior to the WHO post COVID-19 definition, resulting in a heterogeneous delineation of the condition among studies. Lastly, the exclusion of children with PIMS-TS, who typically complain more severe and persistent symptoms, could have an impact on the long COVID prevalence estimation.

## Conclusion

Evidence on long COVID in children is limited, heterogeneous, and based on low-quality studies. Given that an accurate prevalence of the condition remains undefined, it is difficult to distinguish between functional complaints of post-acute COVID syndrome and social restriction effects.

Further high-quality studies are required to define the optimal management of this emergent condition and to establish which resources are needed to face long COVID syndrome and the overall lifelong negative effects of SARS-CoV-2 pandemic in children and adolescents. Since WHO provided a research definition of long COVID, its use should be promoted in future studies to harmonize data. Controlled clinical studies should be encouraged over questionnaire-based ones to ensure an objective analysis of the actual prevalence and long COVID characteristics in children. Moreover, the impact of new variants on long COVID prevalence needs to be investigated to ensure healthcare systems properly allocate their resources.

## Supplementary Information

Below is the link to the electronic supplementary material.

Supplementary file1 (DOCX 30 KB)

## Data Availability

Not applicable.

## Abbreviations

COVID-19: Coronavirus-associated acute respiratory disease called coronavirus disease 19
ECG: Electrocardiography
IQR: Interquartile range
LC28: Long COVID with symptoms persisting over 28 days
LC56: Long COVID with symptoms persisting over 56 days
JBI: Joanna Briggs Institute Critical Appraisal Checklist for Case Series
N: Number
N/A: Not applicable
PIMS-TS: Paediatric inflammatory multisystem syndrome temporally associated with SARS-CoV-2
PRISMA: Preferred Reporting Items for Systematic Reviews and Meta-analyses
SARS-CoV-2: Severe acute respiratory syndrome coronavirus type 2
STROBE: Strengthening the Reporting of Observational Studies in Epidemiology
PCR: Polymerase chain reaction
SD: Standard deviation

## References

[1] Coronaviridae Study Group of the International Committee on Taxonomy of Viruses (2020). The species severe acute respiratory syndrome-related coronavirus: classifying 2019-nCoV and naming it SARS-CoV-2. Nat Microbiol.

[2] Lopez-Leon S, Wegman-Ostrosky T, Perelman C (2021). More than 50 long-term effects of COVID-19: a systematic review and meta-analysis. Sci Rep.

[3] National Istitute for Health and Care Excellence (2020) COVID-19 rapid guideline: managing the long-term effects of COVID-19. http://www.ncbi.nlm.nih.gov/books/NBK567261. Accessed 9 Oct 2021 33555768

[4] CDC (2020) Healthcare workers. In: Cent. Dis. Control Prev. https://www.cdc.gov/coronavirus/2019-ncov/hcp/clinical-care/post-covid-conditions.html. Accessed 9 Oct 2021

[5] Soriano JB, Murthy S, Marshall JC (2021). A clinical case definition of post-COVID-19 condition by a Delphi consensus. Lancet Infect Dis.

[6] Stephenson T, Allin B, Nugawela MD (2022). Long COVID (post-COVID-19 condition) in children: a modified Delphi process. Arch Dis Child.

[7] Galindo R, Chow H, Rongkavilit C (2021). COVID-19 in children. Pediatr Clin North Am.

[8] Henderson LA, Canna SW, Friedman KG (2021). American College of Rheumatology clinical guidance for multisystem inflammatory syndrome in children associated with SARS-CoV-2 and hyperinflammation in pediatric COVID-19: version 2. Arthritis Rheumatol Hoboken NJ.

[9] The BMJ (2020) Counting long covid in children. https://blogs.bmj.com/bmj/2020/10/16/counting-long-covid-in-children/. Accessed 10 Oct 2021

[10] Ludvigsson JF (2021). Case report and systematic review suggest that children may experience similar long-term effects to adults after clinical COVID-19. Acta Paediatr.

[11] Zimmermann P, Pittet LF, Curtis N (2021). How common is long COVID in children and adolescents?. Pediatr Infect Dis J.

[12] Page MJ, McKenzie JE, Bossuyt PM (2021). The PRISMA 2020 statement: an updated guideline for reporting systematic reviews. BMJ.

[13] Say D, Crawford N, McNab S (2021). Post-acute COVID-19 outcomes in children with mild and asymptomatic disease. Lancet Child Adolesc Health.

[14] Sterky E, Olsson-Åkefeldt S, Hertting O (1992). (2021) Persistent symptoms in Swedish children after hospitalisation due to COVID-19. Acta Paediatr Oslo Nor.

[15] von Elm E, Altman DG, Egger M (2007). The Strengthening the Reporting of Observational Studies in Epidemiology (STROBE) statement: guidelines for reporting observational studies. The Lancet.

[16] Munn Z, Barker TH, Moola S (2020). Methodological quality of case series studies: an introduction to the JBI critical appraisal tool. JBI Evid Synth.

[17] Ashkenazi-Hoffnung L, Shmueli E, Ehrlich S (2021). Long COVID in children: observations from a designated pediatric clinic. Pediatr Infect Dis J.

[18] Radtke T, Ulyte A, Puhan MA, Kriemler S (2021). Long-term symptoms after SARS-CoV-2 infection in children and adolescents. JAMA.

[19] Molteni E, Sudre CH, Canas LS (2021). Illness duration and symptom profile in symptomatic UK school-aged children tested for SARS-CoV-2. Lancet Child Adolesc Health.

[20] Miller F, Nguyen V, Navaratnam AM (2021). Prevalence of persistent symptoms in children during the COVID-19 pandemic: evidence from a household cohort study in England and Wales. Pediatrics.

[21] Osmanov IM, Spiridonova E, Bobkova P (2021). Risk factors for long COVID in previously hospitalised children using the ISARIC Global follow-up protocol: a prospective cohort study. Eur Respir J.

[22] Stephenson T, Pereira SMP, Shafran R (2022). Physical and mental health 3 months after SARS-CoV-2 infection (long COVID) among adolescents in England (CLoCk): a national matched cohort study. Lancet Child Adolesc Health.

[23] Smane L, Roge I, Pucuka Z, Pavare J (2021). Clinical features of pediatric post-acute COVID-19: a descriptive retrospective follow-up study. Ital J Pediatr.

[24] Borch L, Holm M, Knudsen M (2022). Long COVID symptoms and duration in SARS-CoV-2 positive children – a nationwide cohort study. Eur J Pediatr.

[25] Heching HJ, Goyal A, Harvey B (2022). Electrocardiographic changes in non-hospitalised children with COVID-19. Cardiol Young.

[26] Roge I, Smane L, Kivite-Urtane A (2021). Comparison of persistent symptoms after COVID-19 and other non-SARS-CoV-2 infections in children. Front Pediatr.

[27] Buonsenso D, Munblit D, De Rose C (2021). Preliminary evidence on long COVID in children. Acta Paediatr.

[28] Brackel CLH, Lap CR, Buddingh EP (2021). Pediatric long-COVID: an overlooked phenomenon?. Pediatr Pulmonol.

[29] Asadi-Pooya AA, Nemati H, Shahisavandi M (2021). Long COVID in children and adolescents. World J Pediatr.

[30] Parisi GF, Diaferio L, Brindisi G (2021). Cross-sectional survey on long term sequelae of pediatric COVID-19 among Italian pediatricians. Children.

[31] Erol N, Alpinar A, Erol C (2022). Intriguing new faces of COVID-19: persisting clinical symptoms and cardiac effects in children. Cardiol Young.

[32] Kikkenborg Berg S, Dam Nielsen S, Nygaard U (2022). Long COVID symptoms in SARS-CoV-2-positive adolescents and matched controls (LongCOVIDKidsDK): a national, cross-sectional study. Lancet Child Adolesc Health.

[33] Zavala M, Ireland G, Amin-Chowdhury Z (2021). Acute and persistent symptoms in children with PCR-confirmed SARS-CoV-2 infection compared to test-negative children in England: active, prospective, national surveillance. Clin Infect Dis Off Publ Infect Dis Soc Am.

[34] Morand A, Campion J-Y, Lepine A (2021). Similar patterns of [18F]-FDG brain PET hypometabolism in paediatric and adult patients with long COVID: a paediatric case series. Eur J Nucl Med Mol Imaging.

[35] Morrow AK, Ng R, Vargas G (2021). Postacute/long COVID in pediatrics: development of a multidisciplinary rehabilitation clinic and preliminary case series. Am J Phys Med Rehabil.

[36] Group IGPC-fuw (2021) ISARIC Global COVID-19 paediatric follow-up. https://isaric.org/research/covid-19-clinical-researchresources/paediatric-follow-up/. Cited 24 Apr 2021

[37] Stephenson T, Shafran R, De Stavola B (2021). Long COVID and the mental and physical health of children and young people: national matched cohort study protocol (the CLoCk study). BMJ Open.

[38] Denina M, Pruccoli G, Scolfaro C (2020). Sequelae of COVID-19 in hospitalized children: a 4-months follow-up. Pediatr Infect Dis J.

[39] Bottino I, Patria MF, Milani GP (2021). Can asymptomatic or non-severe SARS-CoV-2 infection cause medium-term pulmonary sequelae in children?. Front Pediatr.

[40] Knoke L, Schlegtendal A, Maier C et al (2021) More complaints than findings – long-term pulmonary function in children and adolescents after COVID-19. medRxiv 2021.06.22.21259273. 10.1101/2021.06.22.21259273

[41] Bloise S, Isoldi S, Marcellino A (2022). Clinical picture and long-term symptoms of SARS-CoV-2 infection in an Italian pediatric population. Ital J Pediatr.

[42] Yan Z, Yang M, Lai C-L (2021). Long COVID-19 syndrome: a comprehensive review of its effect on various organ systems and recommendation on rehabilitation plans. Biomedicines.

[43] Luijten MAJ, van Muilekom MM, Teela L (2021). The impact of lockdown during the COVID-19 pandemic on mental and social health of children and adolescents. Qual Life Res.

[44] Blankenburg J, Wekenborg M, Reichert J et al (2021) Mental health of adolescents in the pandemic: long-COVID19 or long-pandemic syndrome medRxiv 2021.05.11.21257037. 10.1101/2021.05.11.21257037

[45] Antonelli M, Pujol JC, Spector TD (2022). Risk of long COVID associated with delta versus omicron variants of SARS-CoV-2. Lancet Lond Engl.

[46] Buonsenso D, Piazza M, Boner AL, Bellanti JA (2022). Long COVID: a proposed hypothesis-driven model of viral persistence for the pathophysiology of the syndrome. Allergy Asthma Proc.

[47] Yong SJ (2021). Long COVID or post-COVID-19 syndrome: putative pathophysiology, risk factors, and treatments. Infect Dis Lond Engl.

[48] Izquierdo-Pujol J, Moron-Lopez S, Dalmau J (2022). Post COVID-19 condition in children and adolescents: an emerging problem. Front Pediatr.

[49] Sante GD, Buonsenso D, De Rose C (2021). Immune profile of children with post-acute sequelae of SARS-CoV-2 infection (long COVID).

[50] Schultheiß C, Willscher E, Paschold L (2022). The IL-1β, IL-6, and TNF cytokine triad is associated with post-acute sequelae of COVID-19. Cell Rep Med.

[51] L’Huillier AG, Pagano S, Baggio S (2022). Autoantibodies against apolipoprotein A-1 after COVID-19 predict symptoms persistence. Eur J Clin Invest.

