# Supplementary material for: Prevalence and clinical presentation of long COVID in children: a systematic review
Source: Eur J Pediatr. 2022 Sep 15;181(12):3995–4009. doi: 10.1007/s00431-022-04600-x (PMC9476461; doi:10.1007/s00431-022-04600-x)
Supplement: Supplementary file 1 — Supplementary file1 (DOCX 30 KB) [file 431_2022_4600_MOESM1_ESM.docx]

| 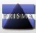**Appendix- Table 1: PRISMA 2020 Checklist** | | | |
| --- | --- | --- | --- |
| **Section and**  **Topic** | **Item**  **#** | **Checklist item** | **Location**  **where item is reported** |
| **TITLE** | | |  |
| Title | 1 | Identify the report as a systematic review. | Page 1 |
| **ABSTRACT** | | |  |
| Abstract | 2 | See the PRISMA 2020 for Abstracts checklist. | Page 1 |
| **INTRODUCTION** | | |  |
| Rationale | 3 | Describe the rationale for the review in the context of existing knowledge. | Page 4 |
| Objectives | 4 | Provide an explicit statement of the objective(s) or question(s) the review addresses. | Page 4 |
| **METHODS** | | |  |
| Eligibility criteria | 5 | Specify the inclusion and exclusion criteria for the review and how studies were grouped for the syntheses. | Page 5 |
| Information  sources | 6 | Specify all databases, registers, websites, organisations, reference lists and other sources searched or consulted to identify studies. Specify the date when each source was last searched or consulted. | Page 5 |
| Search strategy | 7 | Present the full search strategies for all databases, registers and websites, including any filters and limits used. | Page 5 |
| Selection process | 8 | Specify the methods used to decide whether a study met the inclusion criteria of the review, including how many reviewers screened each record and each report retrieved, whether they worked independently, and if applicable, details of automation tools used in the process. | Page 5 |
| Data collection  process | 9 | Specify the methods used to collect data from reports, including how many reviewers collected data from each report, whether they worked independently, any processes for obtaining or confirming data from study investigators, and if applicable, details of automation tools used in the process. | Page 5 |
| Data items | 10a | List and define all outcomes for which data were sought. Specify whether all results that were compatible with each outcome domain in each study were sought (e.g. for all measures, time points, analyses), and if not, the methods used to decide which results to collect. | Page 5 |
|  | 10b | List and define all other variables for which data were sought (e.g. participant and intervention characteristics, funding sources). Describe any assumptions made about any missing or unclear information. | N/A |
| Study risk of bias assessment | 11 | Specify the methods used to assess risk of bias in the included studies, including details of the tool(s) used, how many reviewers assessed each study and whether they worked independently, and if applicable, details of automation tools used in the process. | Page 6 |
| Effect measures | 12 | Specify for each outcome the effect measure(s) (e.g. risk ratio, mean difference) used in the synthesis or presentation of results. | N/A |
| Synthesis  methods | 13a | Describe the processes used to decide which studies were eligible for each synthesis (e.g. tabulating the study intervention characteristics and comparing against the planned groups for each synthesis (item #5)). | Page 5 |
|  | 13b | Describe any methods required to prepare the data for presentation or synthesis, such as handling of missing summary statistics, or data conversions. | Page 5 |
|  | 13c | Describe any methods used to tabulate or visually display results of individual studies and syntheses. | Page 5 |
|  | 13d | Describe any methods used to synthesize results and provide a rationale for the choice(s). If meta-analysis was performed, describe the model(s), method(s) to identify the presence and extent of statistical heterogeneity, and software package(s) used. | N/A |
|  | 13e | Describe any methods used to explore possible causes of heterogeneity among study results (e.g. subgroup analysis, meta-regression). | N/A |
|  | 13f | Describe any sensitivity analyses conducted to assess robustness of the synthesized results. | N/A |
| Reporting bias  assessment | 14 | Describe any methods used to assess risk of bias due to missing results in a synthesis (arising from reporting biases). | Page 5 |
| Certainty  assessment | 15 | Describe any methods used to assess certainty (or confidence) in the body of evidence for an outcome. | Page 5 |

| **RESULTS** | | |  |
| --- | --- | --- | --- |
| Study selection | 16a | Describe the results of the search and selection process, from the number of records identified in the search to the number of studies included in the review, ideally using a flow diagram. | Figure 1 |
|  | 16b | Cite studies that might appear to meet the inclusion criteria, but which were excluded, and explain why they were excluded. | Supplementary materials |
| Study  characteristics | 17 | Cite each included study and present its characteristics. | Table 2 and table 3 |
| Risk of bias in  studies | 18 | Present assessments of risk of bias for each included study. | Figure 2 and figure 3 |
| Results of  individual studies | 19 | For all outcomes, present, for each study: (a) summary statistics for each group (where appropriate) and (b) an effect estimate and its precision (e.g. confidence/credible interval), ideally using structured tables or plots. | Page 6-10  Figure 4-5 |
| Results of  syntheses | 20a | For each synthesis, briefly summarise the characteristics and risk of bias among contributing studies. | Figure 2 and figure 3 |
|  | 20b | Present results of all statistical syntheses conducted. If meta-analysis was done, present for each the summary estimate and its precision (e.g. confidence/credible interval) and measures of statistical heterogeneity. If comparing groups, describe the direction of the effect. | N/A |
|  | 20c | Present results of all investigations of possible causes of heterogeneity among study results. | N/A |
|  | 20d | Present results of all sensitivity analyses conducted to assess the robustness of the synthesized results. | N/A |
| Reporting biases | 21 | Present assessments of risk of bias due to missing results (arising from reporting biases) for each synthesis assessed. | N/A |
| Certainty of  evidence | 22 | Present assessments of certainty (or confidence) in the body of evidence for each outcome assessed. | N/A |
| **DISCUSSION** | | |  |
| Discussion | 23a | Provide a general interpretation of the results in the context of other evidence. | Page 10-13 |
|  | 23b | Discuss any limitations of the evidence included in the review. | Page 13 |
|  | 23c | Discuss any limitations of the review processes used. | Page 13 |
|  | 23d | Discuss implications of the results for practice, policy, and future research. | Page 13 |
| **OTHER INFORMATION** | | |  |
| Registration and protocol | 24a | Provide registration information for the review, including register name and registration number, or state that the review was not registered. | N/A |
|  | 24b | Indicate where the review protocol can be accessed, or state that a protocol was not prepared. | N/A |
|  | 24c | Describe and explain any amendments to information provided at registration or in the protocol. | N/A |
| Support | 25 | Describe sources of financial or non-financial support for the review, and the role of the funders or sponsors in the review. | Page 1 |
| Competing  interests | 26 | Declare any competing interests of review authors. | Page 13 |
| Availability of  data, code and  other materials | 27 | Report which of the following are publicly available and where they can be found: template data collection forms; data extracted from included studies; data used for all analyses; analytic code; any other materials used in the review. | Supplementary materials |

*From:* Page MJ, McKenzie JE, Bossuyt PM, Boutron I, Hoffmann TC, Mulrow CD, et al. The PRISMA 2020 statement: an updated guideline for reporting systematic reviews. BMJ 2021;372:n71. doi: 10.1136/bmj.n71

For more information, visit: <http://www.prisma-statement.org/>

| **Appendix -Table 2: Excluded studies** | **Exclusion criteria** |
| --- | --- |
| Simpson FK, Lokugamage AU. The elephant and the blind men: the children of long covid. BMJ. 2021;372:n157. Published 2021 Jan 19. doi:10.1136/bmj.n157 | No original data |
| Ludvigsson JF. Reporting suspicions of long COVID in children is justified during this global emergency. Acta Paediatr. 2021;110:1373. doi:10.1111/apa.15762 | No original data |
| Thomson H. Children with long covid. New Sci. 2021;249:10-11. doi:10.1016/S0262-4079(21)00303-1 | No original data |
| Stephenson T, Shafran R, De Stavola B, et al. Long COVID and the mental and physical health of children and young people: national matched cohort study protocol (the CLoCk study). BMJ Open. 2021;11:e052838. Published 2021 Aug 26. doi:10.1136/bmjopen-2021-052838 | No prevalence of symptoms reported |
| Hageman JR. Long COVID-19 or Post-Acute Sequelae of SARS-CoV-2 Infection in Children, Adolescents, and Young Adults. Pediatr Ann. 2021;50:e232-e233. doi:10.3928/19382359-20210519-02 | No original data |
| Simpson F, Chew-Graham C, Lokugamage A. Long COVID in children: the perspectives of parents and children need to be heard. Br J Gen Pract. 2021;71(706):216. Published 2021 Apr 29. doi:10.3399/bjgp21X715769 | No original data |
| Altmann DM. Children and the return to school: how much should we worry about covid-19 and long covid?. BMJ. 2021;372:n701. Published 2021 Mar 15. doi:10.1136/bmj.n701 | No original data |
| Bottino I, Patria MF, Milani GP, et al. Can Asymptomatic or Non-Severe SARS-CoV-2 Infection Cause Medium-Term Pulmonary Sequelae in Children?. Front Pediatr. 2021;9:621019. Published 2021 May 13. doi:10.3389/fped.2021.621019 | No prevalence of symptoms reported |
| Ludvigsson JF. Spanish telemedicine data on 8 children support concept of 'long covid' in children. Acta Paediatr. 2021;110:2284. doi:10.1111/apa.15869 | No original data |
| Wise J. Long covid: One in seven children may still have symptoms 15 weeks after infection, data show. BMJ. 2021;374:n2157. Published 2021 Sep 1. doi:10.1136/bmj.n2157 | No original data |
| Dobson CP. Cardiac Sequelae of COVID-19 in Children and Young Adults. Pediatr Ann. 2021;50:e128-e135. doi:10.3928/19382359-20210224-01 | Adults popolutation included |
| Denina M, Pruccoli G, Scolfaro C, et al. Sequelae of COVID-19 in Hospitalized Children: A 4-Months Follow-Up. Pediatr Infect Dis J. 2020;39:e458-e459. doi:10.1097/INF.0000000000002937 | No prevalence of symptoms reported |
| Buonsenso D, Fusco C, De Rose C, Valentini P, Vergari J. Long COVID in children: Partnerships between families and paediatricians are a priority for better care [published online ahead of print, 2021 Jun 1]. J Paediatr Child Health. 2021;10.1111/jpc.15600. doi:10.1111/jpc.15600 | No original data |
| Blankenburg J, Wekenborg M, Reichert J, Kirsten C, Kahre E, Haag L, et al. Mental health of Adolescents in the Pandemic: Long-COVID19 or Long-Pandemic Syndrome [Internet]. medRxiv 2021 [cited 2021 Oct 16]. Available from: https://europepmc.org/article/PPR/PPR338726 | Adults population included |
| Di Sante G, Buonsenso D, De Rose C, Valentini P, Ria F, Sanguinetti M et al. Immune profile of children with post-acute sequelae of SARS-CoV-2 infection (Long Covid) [Internet]. medRxiv 2021.05.07.21256539; doi: https://doi.org/10.1101/2021.05.07.21256539 | No original data |
| Gebhard CE, Sütsch C, Bengs S, Deforth M, Buehler KP, Hamouda N, et al. Sex- and Gender-specific Risk Factors of Post-COVID-19 Syndrome: A Population-based Cohort Study in Switzerland [Internet] medRxiv 2021.06.30.21259757;  doi: https://doi.org/10.1101/2021.06.30.21259757 | Adult population included |
| Magnusson K, Damgaard Skyrud K, Suren P, Greve-Isdahl M, Størdal K, Kristoffersen DT, et al. Health care use up to 6 months after COVID-19 in 700.000 children and adolescents: a pre-post study[Internet] medRxiv 2021.06.02.21258211;  doi: https://doi.org/10.1101/2021.06.02.21258211 | No prevalence of symptoms reported |
| Knoke L, Schlegtendal A, Maier C, et al (2021) More complaints than findings - Long-term pulmonary function in children and adolescents after COVID-19. medRxiv 2021.06.22.21259273; doi: https://doi.org/10.1101/2021.06.22.21259273 | No prevalence of symptoms reported |
